# Independent Controls of Differently-Polarized Reflected Waves by Anisotropic Metasurfaces

**DOI:** 10.1038/srep09605

**Published:** 2015-04-15

**Authors:** Hui Feng Ma, Gui Zhen Wang, Gu Sheng Kong, Tie Jun Cui

**Affiliations:** 1 State Key Laboratory of Millimeter Waves, School of Information Science and Engineering, Southeast University, Nanjing 210096, China

## Abstract

We propose a kind of anisotropic planar metasurface, which has capacity to manipulate the orthogonally-polarized electromagnetic waves independently in the reflection mode. The metasurface is composed of orthogonally I-shaped structures and a metal-grounded plane spaced by a dielectric isolator, with the thickness of about 1/15 wavelength. The normally incident linear-polarized waves will be totally reflected by the metal plane, but the reflected phases of *x*- and *y*-polarized waves can be controlled independently by the orthogonally I-shaped structures. Based on this principle, we design four functional devices using the anisotropic metasurfaces to realize polarization beam splitting, beam deflection, and linear-to-circular polarization conversion with a deflection angle, respectively. Good performances have been observed from both simulation and measurement results, which show good capacity of the anisotropic metasurfaces to manipulate the *x*- and *y*-polarized reflected waves independently.

With the great capacity to manipulate the reflections or refractions of incoming waves, metasurfaces have been attracted more attentions in recent years. The Snell's law provides the refraction and reflection principles when the incoming electromagnetic wave (or light) impinge on the interface of two media with different indexes of refractions. The metasurface has been introduced by applying a series of metallic structures on the interface to generate discontinuous phase shifts, yielding anomalous reflections and refractions, which were explained by the general Snell Law^1^. Based on the metasurface, many interesting works have been presented, such as photonic spin Hall effect^2^, polarization-controlled plasmonic coupler^3^, three-dimensional computer-generated holography image reconstruction^4^, ultrathin flat lenses^5^,^6^, and other applications^7^,^8^. In the meantime, the good asymmetric transmission with cross-polarization conversion also can be achieved by ultra-thin chiral metasurface^9^,^10^.

All above mentioned transmissive metasurfaces have shown exceptional abilities for controlling the refractions of light, but the transmission efficiency is usually low. The reflective metasurfaces constructed by metallic structures placed on the top of a thin dielectric isolator or substrate with grounded plane on the bottom are the other kinds of metasurfaces, which can manipulate the reflections of incoming waves with high efficiency, reaching nearly 100%. The incoming waves can be totally reflected by the grounded plane on the bottom of the isolator or substrate, but the phases of reflected waves can be modulated by the metasurfaces. By designing gradient metallic structures on the top of isolator, the discontinuous phase shifts on the surface can be achieved to modulate the reflected waves. Earlier invetigations have been conducted on the reflective metasurfaces, such as converting the propagating waves to surface waves^11^, focusing mirrors^12^,^13^, anomalous reflections^14^,^15^, and in acoustic application^16^. However, such reflective metasurfaces are isotropic, which have the same responses for both *x*- and *y*-polarized waves, and only a few works based on anisotropic metasurface have been presented^17^,^18^,^19^, which still have limits to control the different polarizations of reflected waves.

In this article, we propose anisotropic reflective metasurfaces, which can manipulate the *x*- and *y*-polarized reflected waves independently with high efficiency. The unit cells of the reflective metasurfaces are a series of orthogonally I-shaped structures, which has anisotropic responses for each of orthogonal polarizations (*x* and *y* polarizations). The normally incident waves are totally reflected by the metal-grounded plane on the bottom of metasurface, but the reflection phases of both *x*- and *y*-polarized waves are controlled independently by changing the dimensions of anisotropic unit cells of metasurface. Based on the proposed anisotropic reflective metasurfaces, four kinds of functional devices are designed, fabricated, and measured for polarization beam splitting, beam deflection, and linear-to-circular polarization conversion with a deflection angle.

## Results

### Theory and design

**Figure 1:**
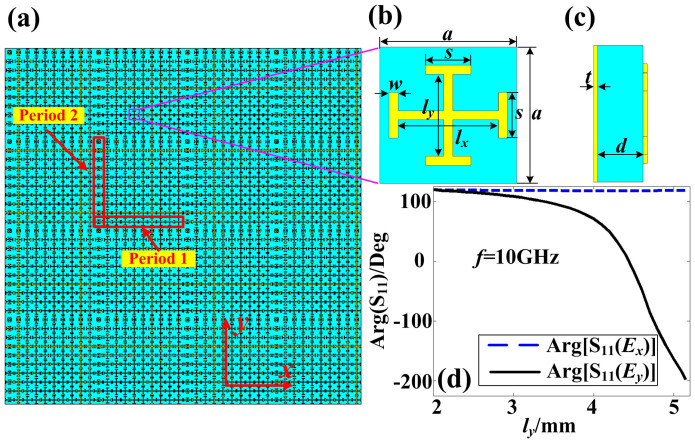
The schematic of birefingent reflective metasurface and its anisotropic unit cell. (a) The schematic of metasurface, which can be composed of only “period 1” or the combination of both “period 1” and “period 2”. (b) The front view of the anisotropic unit cell, which is composed of two orthogonal I-shaped structures. (c) The side view of the anisotropic unit cell, which shows that the metal-grounded plane and the I-shaped structures are spaced by a dielectric isolator. (d) The phase responses of the anisotropic unit cell, which show that the reflection phase of *E_x_* (see blue dashed line) will not be affected by the length changing of *l_y_* but the reflection phase of *E_y_* (see black solid line) is decreased gradually from 130° to −200° when *l_y_* is increased from 2 mm to 5 mm.

The sketch of the reflective metasurface is demonstrated in Fig. 1(a), in which “period 1” and “period 2” are the periods of the metasurface in two orthogonal directions. We remark that the metasurface can be composed of only one kind of period (“period 1” or “period 2”) or the combination of “period 1” and “period 2” according to different applications. The unit cell in each period is orthogonally I-shaped metallic structures, as shown in Fig. 1(b), which are printed on the top of a dielectric substrate. The dimensions of the structures shown in Fig. 1(b) are *a* = 6 mm, *s* = 2 mm, *w* = 0.4 mm, and *l_x_* and *l_y_* are changed independently to control the reflection phases of *x*- and *y*-polarized electromagnetic waves, respectively. On the bottom of the substrate, there is a metal-grounded plane with thickness of *t* = 0.018 mm, and the thickness of the substrate is *d* = 2 mm. The side view of the unit cell is illustrated in Fig. 1(c).

Figure 1(d) demonstrates the reflection phases at 10 GHz for *x*- and *y*-polarized incoming waves with fixed *l_x_* = 3 mm and varied *l_y_* from 2 mm to 5 mm, from which we observe that the reflection phases of the *x*-polarized waves will not be affected, but the reflection phases of the *y*-polarized waves are changed from 130° to −200°. Similarly, if we change the length of *l_x_* from 2 mm to 5 mm with fixed *l_y_*, then the reflection phases of the *x*-polarized waves are changed from 130° to −200°, while the reflection phases of the *y*-polarized waves is not affected. Hence, we conclude that the reflection phases of the *x*- and *y*-polarized waves can be controlled independently by using such orthogonal I-shaped structures by changing the lengths of *l_x_* and *l_y_*, respectively.

Based on the proposed anisotropic reflective metasurface, we present four kinds of functional devices, which have the capacity to manipulate the *x*- and *y*-polarized reflected waves independently. As examples, we design the anisotropic metasurfaces in the microwave frequency, which are realized by printed circuit board (PCB) of F4B with the relative permittivity 2.65 and tangent loss 0.001. The metallic orthogonally I-shaped structures and metal-grounded plane are placed on the top and bottom of thin F4B dielectric with the thickness of *d* = 2 mm. In order to make a clear description of the functions of metasurfaces, a three-dimensional (3D) spherical coordinate system of (*r*, *θ*, *ϕ*) is defined to describe the deflection angle of the reflected waves. The deflection directions of *x*- and *y*-polarized waves are defined as (*ϕ_x_*, *θ_x_*) and (*ϕ_y_*, *θ_y_*), respectively. The relation between the deflection angles of *x*- and *y*-polarized waves in 3D space and the reflection phase-delayed distribution on the metasurface can be derived as
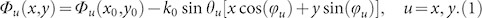
The desired *_u_*(*x*,*y*) can be achieved by the above mentioned gradient metasurface shown in Fig. 1. Meanwhile, the reflected phases of the *x*- and *y*-polarized waves are controlled by changing the lengths of *l_x_* and *l_y_*, respectively. Hence the deflection angles of the reflected *x*- and *y*-polarized waves can be manipulated independently according to Eq. (1).

### Simulation results

**Figure 2:**
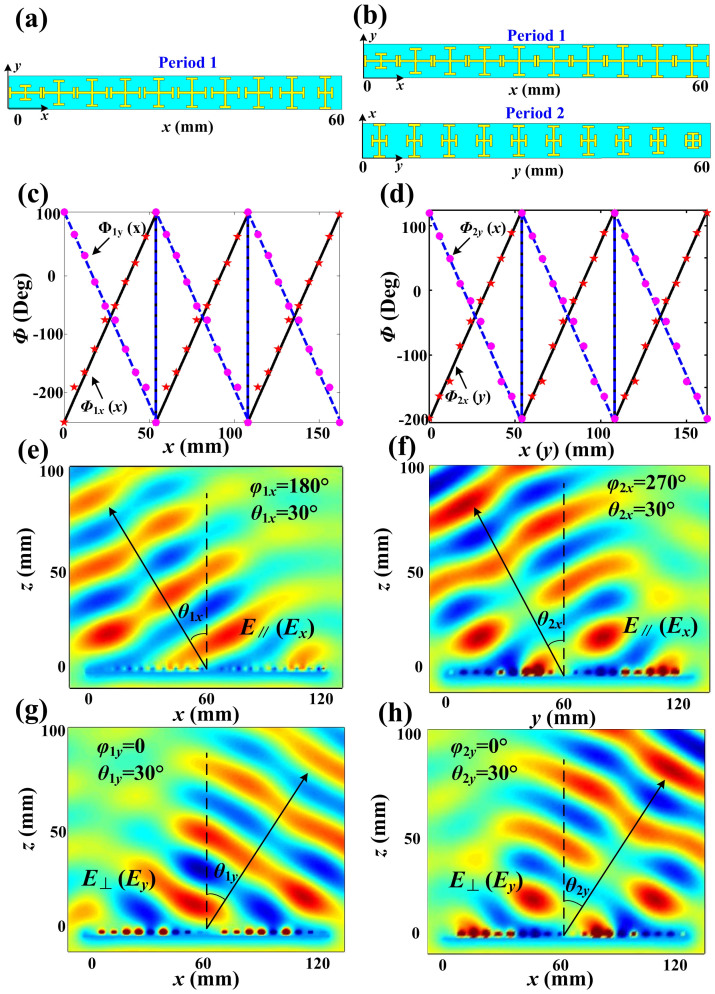
The designing and simulation results of two PBSs. (a, c, e, g) The designing and simulation results of the first PBS: (a) The first PBS is only composed of “period 1”. (c) The phase-delayed distributions of three periods along *x* direction for both *x*- and *y*-polarized reflected waves (*Φ*_1*x*_(*x*) and *Φ*_1*y*_(*x*)), in which solid black line and dashed blue line are calculation results and discrete pentacles and dots are simulation results of actual structures. (e) The electric-field distributions of *x*-polarized reflected waves deflected to (*ϕ*_1*x*_ = 180°, *θ*_1*x*_ = 30°). (g) The electric-field distributions of *y*-polarized reflected waves deflected to (*ϕ*_1*y*_ = 0, *θ*_1*y*_ = 30°). (b, d, f, h) The designing and simulation results of the second PBS: (b) The second PBS is composed of both “period 1” and “period 2”. (d) The phase-delayed distributions of three periods of “period 1” along *x* direction for *y*-polarized reflected waves (*Φ*_2*y*_(*x*)) and three periods of “period 2” along *y* direction for *x*-polarized reflected waves (*Φ*_2*x*_(*y*)), in which solid black line and dashed blue line are calculation results and discrete pentacles and dots are simulation results of actual structures. (e) The electric-field distributions of *x*-polarized reflected waves deflected to (*ϕ*_2*x*_ = 270°, *θ*_2*x*_ = 30°). (g) The electric-field distributions of *y*-polarized reflected waves deflected to (*ϕ*_2*y*_ = 0, *θ*_2*y*_ = 30°).

Based on above discussions, we firstly design two kinds of polarization beam splitters (PBSs) at 10 GHz, as shown in Fig. 2. The first PBS is designed to deflect the *x*- and *y*-polarized reflected waves to the directions of (*ϕ*_1*x*_ = 180°, *θ*_1*x*_ = 30°) and (*ϕ*_1*y*_ = 0, *θ*_1*y*_ = 30°), respectively. The metasurface here is only composed of “period 1”, as illustrated in Fig. 2(a), which is made of ten different unit cells. The lengths of *l_x_* and *l_y_* of the unit cells in one period are increased and decreased gradually along the +*x* direction (*l_x_* = 2 mm, 3.8 mm, 4.2 mm, 4.4 mm, 4.5 mm, 4.65 mm, 4.75 mm, 4.9 mm, 5.05 mm, 5.15 mm; *l_y_* = 5.15 mm, 5.05 mm, 4.9 mm, 4.75 mm, 4.65 mm, 4.5 mm, 4.4 mm, 4.2 mm, 3.8 mm, 2 mm), respectively. According to the relationship between the dimensions of the I-shaped structure and the reflection phase shown in Fig. 1(d), the phase-delayed distributions of three periods along the *x* direction are demonstrated in Fig. 2(c) (see discrete dots and pentacles), which have good agreements with the calculated *Φ*_1*x*_(*x*) and *Φ*_1*y*_(*x*) for (*ϕ*_1*x*_ = 180°, *θ*_1*x*_ = 30°) and (*ϕ*_1*y*_ = 0, *θ*_1*y*_ = 30°) (see the blue dashed line and black solid line in Fig. 2(c), respectively. Figures 2(e) and 2(g) are simulated electric-field distributions of *x*- and *y*-polarized reflected waves. We clearly observe that the *x*- and *y*-polarized reflected waves are deflected to the designed directions of (*ϕ*_1*x*_ = 180°, *θ*_1*x*_ = 30°) and (*ϕ*_1*y*_ = 0, *θ*_1*y*_ = 30°).

The second PBS is designed to deflect the *x*- and *y*-polarized reflected waves to the directions of (*ϕ*_2*x*_ = 270°, *θ*_2*x*_ = 30°) and (*ϕ*_2*y*_ = 0°, *θ*_2*y*_ = 30°), respectively. Both “period 1” and “period 2” are used to construct the metasurface, as shown in Fig. 2(b), in which *l_y_* is increased gradually but *l_x_* is unchanged along the +*x* direction in “period 1” (*l_x_* = 5.15 mm and *l_y_* = 2 mm, 3.8 mm, 4.2 mm, 4.4 mm, 4.5 mm, 4.65 mm, 4.75 mm, 4.9 mm, 5.05 mm, 5.15 mm); while *l_x_* is decreased gradually but *l_y_* is unchanged along the +*y* direction in “period 2” (*l_x_* = 5.15 mm, 5.05 mm, 4.9 mm, 4.75 mm, 4.65 mm, 4.5 mm, 4.4 mm, 4.2 mm, 3.8 mm, 2 mm and *l_y_* = 2 mm). The phase-delayed distributions of three periods for both *Φ*_2*x*_(*y*) and *Φ*_2*y*_(*x*) along the +*x* and +*y* directions are demonstrated in Fig. 2(d) (see the discrete dots and pentacles), which show good agreements with the calculation results (see the blue dashed line and black solid line). The simulation results of electric-field distributions are given in Figs. 2(f) and 2(h). We notice that the *x*-polarized reflected waves are deflected to the direction of (*ϕ*_2*x*_ = 180°, *θ*_2*x*_ = 30°), and the *y*-polarized reflected waves are deflected to (*ϕ*_2*y*_ = 270°, *θ*_2*y*_ = 30°). From the simulation results of above two kinds of PBSs, we conclude that the *x*- and *y*-polarized reflected waves can be separated and deflected independently in the 3D space, showing good agreements with the theoretical analyses.

**Figure 3:**
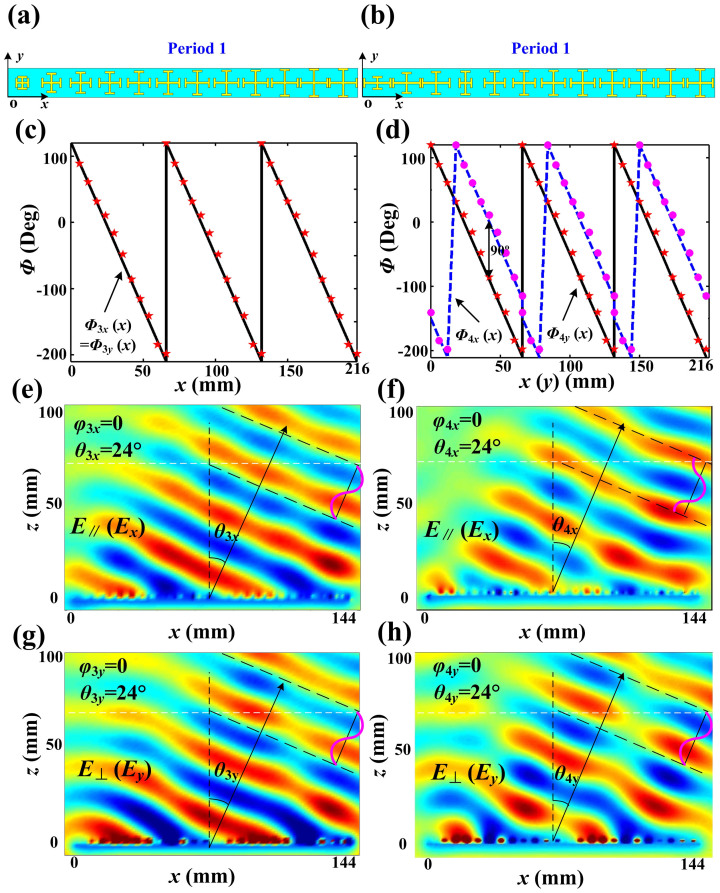
The designing and simulationd results of beam deflection and polarization conversion with a deflection angle. (a, c, e, g) The designing and simulation results of the beam deflection: (a) The third metasurface of beam deflection is only made up of “period 1”. (c) The phase-delayed distributions of three periods along *x* direction for both *x*- and *y*-polarized reflected waves (*Φ*_3*x*_(*x*) and *Φ*_3*y*_(*x*)), in which solid black line is calculationd result and discrete pentacles are simulation results of actual structures. (e) The electric-field distributions of *x*-polarized reflection waves deflected to (*ϕ*_3*x*_ = 0, *θ*_3*x*_ = 24°). (g) The electric-field distributions of *y*-polarized reflection waves deflected to (*ϕ*_3*y*_ = 0, *θ*_3*y*_ = 24°), whose reflection phase is the same with that of *x*-polarized waves. (b, d, f, h) The designing and the simulation results of the linear-to-circular polarization conversion with a deflection angle: (b) The third metasurface of beam deflection is also only made of “period 1”. (d) The phase-delayed distributions of three periods along *x* direction for both *x*- and *y*-polarized reflection waves (*Φ*_4*x*_(*x*) and *Φ*_4*y*_(*x*)), in which solid black line and dashed blue line are calculation results and discrete pentacles and dots are simulation results of actual structures. (f) The electric-field distributions of *x*-polarized reflection waves deflected to (*ϕ*_4*x*_ = 0, *θ*_4*x*_ = 24°). (h) The electric-field distributions of *y*-polarized reflection waves deflected to (*ϕ*_4*y*_ = 0, *θ*_4*y*_ = 24°), whose reflection phase is 90° ahead compared to *y*-polarized waves.

The metasurface can also be designed to deflect the *x*- and *y*-polarized reflected waves to the same direction, but the phases of two orthogonal polarizations can be controlled as desired. As example, the third metasurface deflects the reflected beam to a designed angle, which has the capacity to deflect *x*- and *y*-polarized reflected waves to the same direction with the same phase. In this case, the metasurface is only composed of “period 1” with 12 unit cells, as shown in Fig. 3(a). *l_x_* and *l_y_* of the unit cell are equal and increased gradually along the +*x* direction in “period 1” (*l_x_* = *l_y_* = 2 mm, 3.7 mm, 4.1 mm, 4.3 mm, 4.45 mm, 4.5 mm, 4.6 mm, 4.75 mm, 4.8 mm, 4.95 mm, 5.1 mm, 5.15 mm). The reflected phase-delayed distributions of three periods for *Φ*_3*x*_(*x*) and *Φ*_3*y*_(*x*) along the +*x* direction are demonstrated in Fig. 3(c), in which the simulation results (see discrete dots) and calculation results (see black solid line) show very good agreements. The simulated electric-field distributions are presented in Figs. 3(e) and 3(g), in which both the *x*- and *y*-polarized waves are deflected to the direction of (0, 24°) with the same phase distributions.

The fourth metasurface can convert linear-polarized waves to circular-polarized waves with a deflection angle, which has capacity to deflect both *x*- and *y*-polarized reflected waves to the same direction with the phase difference of 90°. Here, the metasurface is only constructed by “period 1” with 12 unit cells, as shown in Fig. 3(b). The lengths of *l_x_* and *l_y_* of the unit cell are designed differently and gradually changed along the *x* direction (*l_x_* = 4.95 mm, 5.1 mm, 5.15 mm, 2 mm, 3.7 mm, 4.1 mm, 4.3 mm, 4.45 mm, 4.5 mm, 4.6 mm, 4.75 mm, 4.8 mm; *l_y_* = 2 mm, 3.7 mm, 4.1 mm, 4.3 mm, 4.45 mm, 4.5 mm, 4.6 mm, 4.75 mm, 4.8 mm, 4.95 mm, 5.1 mm, 5.15 mm) to make sure that the reflected phase difference of each unit cell for the *x*- and *y*-polarized waves is 90°. Figure 3(d) illustrates the reflected phase-delayed distributions of three periods for *Φ*_4*x*_(*x*) and *Φ*_4*y*_(*x*) along the +*x* direction. The simulated electric-field distributions are given in Figs. 3(f) and 3(h), from which we observe that both the *x*- and *y*-polarized waves are deflected to the same direction (0, 24°), but the phase of the *x*-polarized waves is 90° ahead to the *y*-polarized waves. Hence, the linear-polarized incoming waves with normal incidence are reflected by the metasurface to the direction of (0, 24°) with the circular polarization.

### Experimental results

**Figure 4:**
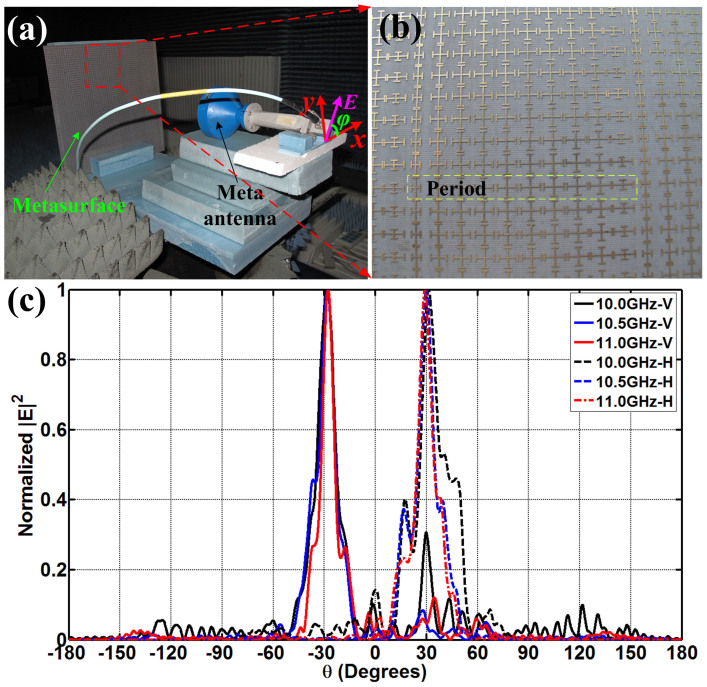
The measurement of the first PBS. (a) The experiment setup, in which an X-band metamaterial lens antenna is used as an excitation to generate the linear-polarized incident plane waves. (b) The photograph of fabricated metasurface. (c) The measured far-field patterns, in which the solid lines are vertical (*y*) polarizations and dashed lines are horizontal (*x*) polarizations.

We have fabricated and measured the first PBS and the linear-to-circular polarization converter, whose periodic arrays are demonstrated in Figs. 2(a) and 3(b), respectively. The designed PBS is to separate the *x*- and *y*-polarized reflected waves to the directions (*ϕ*_1*x*_ = 180°, *θ*_1*x*_ = 30°) and (*ϕ*_1*y*_ = 0, *θ*_1*y*_ = 30°), respectively, whose simulated electric-field distributions are shown in Figs. 2(e) and 2(g). In measurements, the metasurface is placed in an anechoic chamber, and an X-band metamaterial lens antenna^20^ is used to generate the linearly-polarized planar incident waves (see Fig. 4(a)), which can transform quasi spherical waves to planar waves directly. The metamaterial lens is fed by a rectangular waveguide, which is connected to the Vector Network Analyzer (VNA, Agilent N5230C) via a cable. The rectangular waveguide here only supports the dominant TE_10_ mode, whose electric-field vectors are vertical to the wide side of the waveguide. We place the X-band metamaterial lens antenna in front of the metasurface and rotate the lens antenna with an *ϕ*_0_ = 45° angle, as shown in Fig. 4(a), to make sure the electric-field vector of incident planar waves are polarized along the angle *ϕ*_0_ = 45° with respect to the +*x* axis. Hence the total incident electric-field vector (**E**) can be decomposed to **E***_x_* and **E***_y_* components equally.

The experimental setup shown in Fig. 4(a) is placed on a cloud terrace, which can be rotated from −180° to +180° in the horizontal plane. An X-band standard rectangular horn is fixed in another side of the anechoic chamber as receiver, which can be put with horizontally or vertically polarized to receive the *x*- and *y*-polarized reflected waves, respectively. Figure 4(b) depicts the photograph of the metasurface, which is fabricated by F4B with thickness of 2 mm. In Fig. 4(c), the measured far-field patterns demonstrate that the *x*-polarized reflected waves (see the blue solid line) are deflected to the direction −30° and the *y*-polarized reflected waves (see the black solid line) are deflected to the direction +30° from 10 GHz to 11 GHz, which have good agreements with the previous numerical simulations.

**Figure 5:**
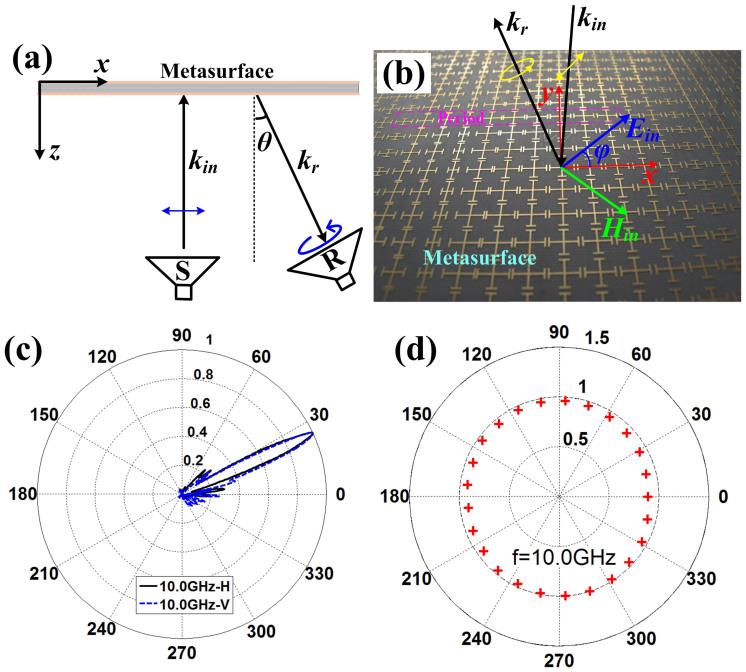
The measurement of the linear-to-circular polarization conversion with a deflection angle. (a) The sketch of the measurement system. A linear-polarized horn antenna is used as excitation to generate linear-polarized normally incident waves, and another linear-polarized horn antenna is placed having an angle of *θ* with metasurface as receiver to receive the circular-polarized reflected waves by rotating 360 degrees of the horn. (b) The photograph of the fabricated metasurface. (c) The measured far-field patterns of the reflected waves at 10 GHz, which show that both horizontal (*x*) and vertical (*y*) polarized waves are all deflected to *θ* = 24°. (d) The measured polarization pattern of the reflected waves at 10 GHz.

The linear-to-circular polarization converter can transform the linear-polarized normally incoming waves to circular-polarized reflected waves with deflection angle of (0, 24°). For this purpose, the sketch of experimental setup is demonstrated in Fig. 5(a), in which two antennas are placed in front of the metasurface to transmit the normally incident plane waves and receive the deflected reflection waves. We notice that the aperture of transmitting antenna (X-band metamaterial lens antenna) is fixed to parallel with the metasurface to generate the normally incident plane waves, and the aperture of receiving antenna (standard X-band horn) is placed with angle *θ* to receive the deflected reflection waves, which can be rotated 360° along its symmetrical axis. The electric vector of linear-polarized incident waves should be polarized along the angle *ϕ* = 45° to obtain equal *E_x_* and *E_y_*, as shown in Fig. 5(b). Hence the reflected *E_x_* and *E_y_* can be controlled by the anisotropic gradient metasurface independently to make the beams of *E_x_* and *E_y_* deflected to the same direction but have 90° phases difference to achieve circular-polarized reflected waves.

Before measuring the polarization pattern, we first measured the far-field radiation patterns of the reflected waves to conform the deflecting angle, as shown in Fig. 5(c). We clearly see that both *x*- and *y*-polarized reflected waves are deflected to the direction (*θ* = 24°) at 10 GHz. Then we put the receiving horn in front of the metasurface with angle *θ* = 24° to receive the reflected waves, and the polarization pattern is achieved by rotating 360° of the linearly-polarized receiving horn, as demonstrated in Fig. 5(d). The axial ratio (AR) of the measured circular polarization is calculated by the definition *AR* = 20log(*b*/*a*) quantitatively, in which *a* and *b* represent the lengths of short and long axes of an ellipse, respectively. As a result, *AR* = 1 dB is achieved, which means that the linearly-polarized normally incident waves are converted to circular-polarized reflected waves with good performance.

## Discussion

In this article, we presented a kind of anisotropic reflective metasurface to control different polarizations of reflected waves. The metasurface is a sandwich structure composed of a series of anisotropic unit cells and a metal-grounded plane spaced by a dielectric substrate of F4B, and the thickness of the metasurface is only about 1/15 wavelength. The normally linearly-polarized incident plane waves can be totally reflected by the metasurfce, but the reflected phases of both *x*- and *y*-polarized waves are controlled independently by the anisotropic metallic unit cells on the top of measurface. Based on this idea, four kinds of functional metasurfaces have been designed to realize two PBSs, a beam deflection and a linear-to-circular polarization conversion with a deflection angle. The simulation results show good performance as theoretical expectation, and the first PBS and linear-to-circular polarization converter are designed and fabricated, whose measurment results show good agreements with the simulations. Hence, we can conclude that the proposed birefringnet metasurfaces have good capacity to manipulate the *x*- and *y*-polarized reflected waves independently with high efficiency.

## Author Contributions

H.F.M. designed, performed, interpreted the experiments and wrote manuscript. G.Z.W. generated numerical simulations and interpreted the experiments. G.S.K. interpreted the experiments. T.J.C. supervised and interpreted the design and experiments, and wrote the manuscript.
